# Impact of healthcare-associated infections on in-hospital outcomes during the COVID-19 era: a multicenter comparative study of 20,942 isolated microorganisms from ICU patients

**DOI:** 10.3389/fpubh.2025.1475221

**Published:** 2025-02-07

**Authors:** Armin Khavandegar, Zeinab Siami, Aziz Rasouli, Pershang Nazemi, Anoosha Gull

**Affiliations:** ^1^Sina Trauma and Surgery Research Center, Tehran University of Medical Sciences, Tehran, Iran; ^2^Department of Infectious Disease and Tropical Medicine, Ziaeian Hospital, Tehran University of Medical Sciences, Tehran, Iran; ^3^Department of Emergency Medicine, School of Medicine, Ziaeian Hospital, Tehran University of Medical Sciences, Tehran, Iran; ^4^Infectious Diseases Department, Yas Hospital Complex, Tehran University of Medical Sciences, Tehran, Iran; ^5^Student Research Committee, School of Medicine, Tehran University of Medical Sciences, Tehran, Iran

**Keywords:** intensive care unit (ICU), healthcare-associated infection (HAI), nosocomial infection, infection prevention and control (IPC), coronavirus disease 2019 (COVID-19), pandemic-related infections, urinary tract infections (UTI), surgical site infection (SSI)

## Abstract

**Background:**

Healthcare-associate infection (HAI) has been one of the health care issues worldwide. During the COVID-19 pandemic, HAI prevention was considered a vital aspect of the clinical management of COVID-19. In the present study, we aimed to compare the frequency of HAIs in ICU-admitted cases within and before the COVID-19 era.

**Methods:**

This multicenter retrospective study applied a descriptive-analytical approach to investigate the frequency of HAIs in the ICU departments of hospitals affiliated with Tehran University of Medical Sciences from 2017 to 2022.

**Results:**

Overall, 14,268 cases with 20,942 isolated microorganisms were recruited for this study, with 8,289 (58.1%) of them being male and 5,975 (41.9%) being female. A total of 6,722 (47.1%) cases with 9,917 (47.4%) isolated microorganisms were documented in the pre-COVID-19 era, while 7,546 (52.9%) patients with 11,025 (52.64%) were reported in COVID-19 era. Of 14,268 patients admitted to the ICU during the study period, 9,197 (64.8%) were discharged and 5,071 died (35.5%), of which 1,970 (29.3%) occurred in pre-COVID and 4,752 (70.7%) in the COVID-19 era (*p*-value < 0.001). *Klebsiella* species, *Acinetobacter* species, *Escherichia coli*, *Staphylococcus* species, and *Pseudomonas aeruginosa* were the five most frequent isolated microorganisms, reported in 5,542 (26.50%), 4,171 (19.90%), 2,651 (12.70%), 2,537 (12.10%), and 1,851 (8.80%) cultures, respectively. VAEs were the most common infection types as reported in 3,562 (25%) patients. There was a statistically significant difference in UTI, PMEU, BSI, and others, with an increase in PMEUs (13.5% vs. 17.1%) and BSI (22.7% vs. 24.5%) and a decrease in UTIs (24.5% vs. 22.2%) and others (4.2% vs. 2.6%) in COVID-19 era compared to pre-COVID-19. The odds of in-hospital mortality in all six main infection categories have significantly increased in the COVID-19 era when compared to the pre-COVID-19 era. The odds of death were increased in the SSI group more than the rest (OR:2.65, CI 95%: 2.13–3.29).

**Conclusion:**

COVID-19 changed the pattern of HAIs and also increased their mortality. Overall, the findings of this study emphasize the importance of continuous monitoring and improvement of infection control measures in ICUs to reduce the incidence of HAIs and improve patient outcomes.

## 1 Introduction

A Healthcare-Associated Infection (HAI), defined as an infection occurring on or after the third day of a patient’s hospital admission (1), has been one of the health care issues around the world (1, 2). The most common HAIs are surgical site infections, urinary tract infections, pneumonia, and bloodstream infections (3). HAIs in intensive care units (ICUs) are among the avoidable morbidity and mortality causes that also lead to increased hospital length of stay (LOS), associated costs, and multiple antibiotic resistance, especially in developing countries (2).

Finding from the European Prevalence of Infection in Intensive Care (EPIC) Study highlights a concerning issue within ICUs across Europe, with a nosocomial infection prevalence of approximately 20.6%, it is evident that these infections pose a significant challenge to patient safety and healthcare systems (4). The European Centre for Disease Prevention and Control (ECDC) Annual Epidemiological Report for 2019 revealed that 7.4% of ICU patients who stayed for more than 2 days amounting to 8,874 out of 120,446 individuals developed at least one HAI (5). Moreover, in 2020, the ECDC highlighted how the COVID-19 pandemic exacerbated HAI rates in ICUs. The incidence of HAIs was notably higher in ICUs treating COVID-19 patients, reaching 16.59 infections per 1,000 patient-days, compared to 13.42 infections per 1,000 patient-days in ICUs without COVID-19 cases during the same period (6).

Invasive devices such as endotracheal tubes, central venous catheters, and urinary catheters play a crucial role in the management of critically ill patients in the ICU. However, their use also comes with inherent risks, including the potential for infections (7). The fact that up to a third of these infections may be preventable highlights the need for improved infection control practices within ICUs (8). The consequences of these infections cannot be understated. Each infection not only poses a direct threat to patient health but also leads to increased mortality rates. The compromised immune systems of ICU patients make them more susceptible to severe infections, which can quickly escalate into life-threatening conditions if not promptly treated (9).

During the COVID-19 pandemic, the prevention of HAIs was considered a vital aspect in the clinical management of COVID-19 because hospital-acquired coinfection, mainly attributed to bacteria, poses a worrying challenge in the context of COVID-19 and how it is handled (10). The presence of microbial coinfection poses challenges for COVID-19 patients as it adds complexity to the initial viral infection, worsens the outcome, and significantly raises the risk of mortality (11, 12). It has been firmly established that unstable COVID-19 cases, especially ICU admitted cases, exhibit a greater incidence of microbial and, particularly, fungal coinfection (13).

Considering that the prevention of HAIs, especially in ICU admitted cases is currently a global priority, and accurate knowledge of its rate and incidence at the national or international level using reliable data has led to appropriate measures to control infection and reduce the incidence of infection in hospitals. In the present multicenter study, firstly, we aimed to compare the frequency of HAIs in ICU-admitted cases within and before the COVID-19 era. Then, we made a comparison of two main outcomes, i.e., in-hospital mortality and prolonged ICU LOS, in each infection category between the pre-COVID-19 and COVID-19 era.

## 2 Materials and methods

### 2.1 Study design

This multicenter retrospective study applied a descriptive-analytical approach to investigate the frequency of HAIs in the ICU departments of hospitals affiliated with Tehran University of Medical Sciences hospitals from 21st March 2017 to 22nd September 2022. Data were extracted from the hospital infection care software system, during the COVID-19 pandemic and pre-COVID era. Eventually, a comparison of HAIs frequency was made between these two. Confirmation and diagnosis of HAIs were based on the ECDC guideline published in 2022 and Center for Disease Control and Prevention (CDC) published in 2024. Theses guideline, similar to previous versions, defines HAIs as infections occurring on or after the third calendar day of a patient’s hospital admission (1, 2). Patients younger than 18 years were excluded from this study. This study is approved by the Research Ethics Committee of Tehran University of Medical Sciences with Approval ID: IR.TUMS.IKHC.REC.1401.356.

### 2.2 Medical history, laboratory measurement, and outcomes

The study included all patients admitted to the ICUs of Tehran University of Medical Sciences hospitals from 2017 to 2022. After the first 48 h of admission and within the first 3 days of being admitted to the ICU, various specimens such as sputum, endotracheal aspirates, nasopharyngeal and oropharyngeal swabs were gathered, employed containers meeting the CDC recommended guidelines for collecting, transporting, and processing specimens (14). Both respiratory and blood culture specimens were dispatched to the laboratory.

Bacterial pathogens were primarily identified using classical culture methods on standard media (e.g., blood agar, MacConkey agar, and chocolate agar). Identification was further confirmed using an automated biochemical identification system (VITEK 2). Furthermore, fungal species, including Candida species, were initially differentiated using chromogenic agar. Further species-level identification of fungi and confirmation of less commonly isolated pathogens (e.g., Aspergillus and Mucormycosis species) were performed using MALDI-TOF MS (Matrix-Assisted Laser Desorption/Ionization Time-of-Flight Mass Spectrometry).

Diabetic Mellitus, Hypertension, Ischemic heart disease, cancer, renal and hepatic disorders, and neurological disorders were underlying diseases. The selection of these comorbidities was made based on expert clinicians’ opinions. A solar calendar was utilized to report isolated microorganisms each year. An equivalent Gregorian calendar was also utilized for data presentation. Two main outcomes were assessed in the current study, in-hospital mortality and prolonged ICU LOS. Prolonged ICU admission was defined as ICU LOS ≥ 10 days. In the present study, we categorized all infection types into six main categories; ventilator-associated events (VAE), bloodstream infections (BSI), urinary tract infections (UTI), pneumonia with extrapulmonary involvement (PMEU), surgical site infections (SSI), and others.

### 2.3 Statistical analysis

Quantitative variables were reported as mean and standard deviation or median and interquartile range. Nominal and categorical data are presented as frequency (%). Statistical comparisons of numbers and percentages between two groups were performed using the chi-square test. To check the relationship between other variables before and after the COVID-19 pandemic, based on the normality test and the type of variable (quantitative, qualitative), paired t-test, McNemar or Wilcoxon were used. All statistical analyses were conducted by SPSS version 27. A *p*-value of less than 0.05 is considered statistically significant in all analyses. Adjusted Bonferroni was employed to compare numbers (percentages) between groups. Univariable and multiple logistic regression models were used to compare the impact of COVID-19 on two main outcomes based on each infection category.

## 3 Results

Overall, 14,268 ICU admitted patients in pre-pandemic and pandemic periods with 20,942 isolated microorganisms were recruited for this study. A total of 6,722 (47.1%) cases with 9,917 (47.4%) isolated microorganisms were documented in the pre-COVID-19 era, while 7,546 (52.9%) patients with 11,025 (52.64%) isolated microorganisms were reported in COVID-19 era.

**Figure 1:**
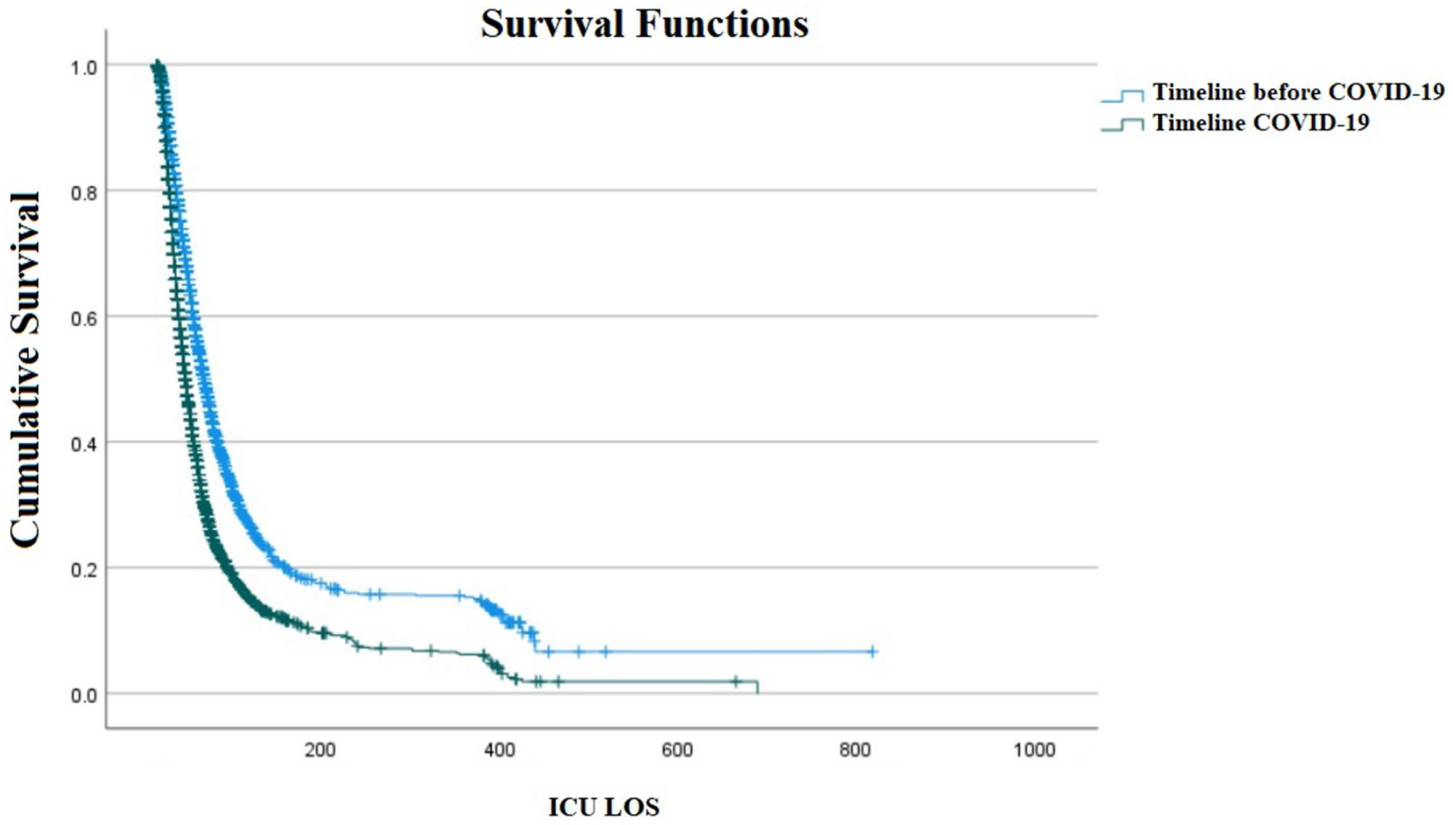
Kaplan–Meier survival plot of ICU admitted patients with confirmed Healthcare-Associated Infections (HAIs) between COVID-19 and pre-COVID-19 era (*p* < 0.001).

A total of 8,289 (58.1%) of them being male and 5,975 (41.9%) being female. The gender was unknown in 4 (0%) cases. Overall, 7,368 cases (51.6%) had underlying diseases. Of 14,268 patients admitted to the ICU during the study period, 9,197 (64.8%) were discharged and 5,071 died (35.5%), of which 1,970 (29.3%) death occurred in pre-COVID era and 4,752 (70.7%) during the COVID-19 era (*p*-value < 0.001). Furthermore, a total of 9,321 (84.63%) patients had a prolonged ICU LOS, with 4,565 (49%) in the pre-COVID era and 4,756 (51%) during COVID-19 (*p* = 0.952). the median (IQR) LOS was 23 (28) in pre-COVID era and 21 (23) in COVID-era. As demonstrated in Figure 1, there was a statistically significant difference in ICU-admitted patients with confirmed HAIs between COVID-19 and the pre-COVID-19 era (*p* < 0.001).

As demonstrated in Table 1, the most frequent number of isolated microorganisms was documented in March 2021 to March 2022 with 4,407 (21.04%), followed by 4,067 (19.42%) in March 2020 to March 2021, 4,042 (19.30%) in March 2017 to March 2018, 3,917 (18.70%) in March 2019 to March 2020, 2,251 (10.75%) in March 2022 to September 2022, and 1,958 (9.34%) in March 2018 to March 2019. *Klebsiella* species, *Acinetobacter* species, *Escherichia coli*, *Staphylococcus* species, and *Pseudomonas aeruginosa* were the five most frequent isolated microorganisms, reported in 5,542 (26.50%), 4,171 (19.90%), 2,651 (12.70%), 2,537 (12.10%), and 1,851 (8.80%) cultures, respectively. *Candida* species, *Aspergillus* species, and *Mucormycosis* species were the only isolated fungal microorganisms, with a frequency of 994 (4.70%), 2 (0%), and 2 (0%), respectively.

**Table 1:** Number of isolated microorganisms in each year in order of their frequency.

Timeline	Solar system	1,396	1,397	1,398	1,399	1,400	1,401	Total
	Gregorian system	March 2017 to March 2018	March 2018 to March 2019	March 2019 to March 2020	March 2020 to March 2021	March 2021 to March 2022	March 2022 to September 2022	
*Klebsiella* spp.		852 (21.10)	413 (21.10)	1,031 (26.30)	1,182 (29.10)	1,330 (30.20)	734 (28.80)	5,542 (26.50)
*Acinetobacter* spp.		692 (17.10)	288 (14.70)	796 (20.30)	958 (23.60)	902 (20.50)	535 (21.00)	4,171 (19.90)
*Escherichia coli*		572 (14.20)	264 (13.50)	517 (13.20)	509 (12.50)	486 (11.00)	303 (11.90)	2,651 (12.70)
*Staphylococcus* spp.		610 (15.10)	326 (16.60)	526 (13.40)	423 (10.40)	434 (9.80)	218 (8.50)	2,537 (12.10)
*Pseudomonas aeruginosa*		348 (8.60)	152 (7.80)	344 (8.80)	319 (7.80)	418 (9.50)	270 (10.60)	1,851 (8.80)
*Enterococcus* spp.		266 (6.60)	122 (6.20)	216 (5.50)	270 (6.60)	359 (8.10)	181 (7.10)	1,414 (6.80)
*Candida* spp.		320 (7.90)	136 (6.90)	181 (4.60)	106 (2.60)	125 (2.80)	126 (4.90)	994 (4.70)
*Enterobacter* spp.		137 (3.40)	109 (5.60)	143 (3.70)	157 (3.90)	201 (4.60)	82 (3.20)	829 (4.00)
*Streptococcus* spp.		70 (1.70)	32 (1.60)	37 (0.90)	39 (1.00)	32 (0.70)	17 (0.70)	227 (1.10)
*Stenotrophomonas maltophilia*		37 (0.90)	62 (3.20)	28 (0.70)	30 (0.70)	42 (1.00)	27 (1.10)	226 (1.10)
*Proteus* spp.		48 (1.20)	18 (0.90)	47 (1.20)	29 (0.70)	27 (0.60)	23 (0.90)	192 (0.90)
*Citrobacter* spp.		43 (1.10)	7 (0.40)	23 (0.60)	19 (0.50)	29 (0.70)	12 (0.50)	133 (0.60)
*Sarashia* spp.		42 (1.00)	23 (1.20)	18 (0.50)	16 (0.40)	12 (0.30)	9 (0.40)	120 (0.60)
*Burkholderia* spp.		0 (0)	4 (0.20)	1 (0)	2 (0)	5 (0.10)	7 (0.30)	19 (0.10)
*Salmonella* spp.		1 (0)	0 (0)	3 (0.10)	2 (0)	1 (0)	1 (0)	8 (0)
*Micrococcus* spp.		1 (0)	0 (0)	2 (0.10)	1 (0)	2 (0)	2 (0.10)	8 (0)
*Bacillus anthracis*		0 (0)	0 (0)	1 (0)	2 (0)	0 (0)	0 (0)	3 (0)
*Shigella* spp.		1 (0)	2 (0.10)	0 (0)	0 (0)	0 (0)	0 (0)	3 (0)
*Clostridium perfringens*		0 (0)	0 (0)	0 (0)	0 (0)	0 (0)	3 (0.10)	3 (0)
*Clostridium difficile*		0 (0)	0 (0)	1 (0)	0 (0)	0 (0)	1 (0)	2 (0)
*Corynebacterium diphtheria*		1 (0)	0 (0)	1 (0)	0 (0)	0 (0)	0 (0)	2 (0)
*Morganella* spp.		0 (0)	0 (0)	1 (0)	0 (0)	1 (0)	0 (0)	2 (0)
*Aspergillus* spp.		0 (0)	0 (0)	0 (0)	1 (0)	1 (0)	0 (0)	2 (0)
*Mucormycosis* spp.		0 (0)	0 (0)	0 (0)	2 (0)	0 (0)	0 (0)	2 (0)
*Bacillus cereus*		1 (0)	0 (0)	0 (0)	0 (0)	0 (0)	0 (0)	1 (0)
Total		4,042	1,958	3,917	4,067	4,407	2,551	20,942

Numbers are presented as *N* (%).

As presented in Table 2, VAEs were the most common infection types as reported in 3,562 (25%) patients, followed by BSIs in 3,380 (23.7%), UTIs in 3,327 (23.3%), PMEUs in 2,196 (15.4%), SSIs in 1,326 (9.3%), and others in 477 (3.3%). *Acinetobacter* species were the most frequently reported isolated bacteria in VAEs (*n* = 1,784, 33.4%), *Klebsiella* species in BSIs (*n* = 1,152, 23.2%), *Escherichia coli* in UTIs (*n* = 1,550, 31.2%), *Acinetobacter* species in PMEUs (*n* = 1,133, 36.6%), *Klebsiella* species in SSIs (*n* = 438, 22.9%), and *Staphylococcus* species in Others category (*n* = 179, 28.6%).

**Table 2:** Frequency of isolated microorganisms based on six main infection categories in order of their frequency.

	VAE	BSI	UTI	PMEU	SSI	Others	Total
Number of patients in each group; *n* (%)	3,562 (25)	3,380 (23.7)	3,327 (23.3)	2,196 (15.4)	1,326 (9.3)	477 (3.3)	14,268 (100)
Total number of microorganisms in each group; *n* (%)	5,340 (25.5)	4,972 (23.7)	4,970 (23.7)	3,122 (14.9)	1,912 (9.1)	626 (3)	20,942
*Klebsiella* spp.	1,759 (32.9%)	1,152 (23.2%)	1,236 (24.9%)	829 (26.6%)	438 (22.9%)	128 (20.4%)	5,542 (26.50)
*Acinetobacter* spp.	1,784 (33.4%)	651 (13.1%)	252 (5.1%)	1,144 (36.6%)	270 (14.1%)	70 (11.2%)	4,171 (19.90)
*Escherichia coli*	309 (5.8%)	334 (6.7%)	1,550 (31.2%)	169 (5.4%)	219 (11.5%)	70 (11.2%)	2,651 (12.70)
*Staphylococcus* spp.	353 (6.6%)	1,136 (22.8%)	133 (2.7%)	307 (9.8%)	429 (22.4%)	179 (28.6%)	2,537 (12.10)
*Pseudomonas aeruginosa*	584 (10.9%)	350 (7.0%)	397 (8.0%)	314 (10.1%)	149 (7.8%)	57 (9.1%)	1,851 (8.80)
*Enterococcus* spp.	33 (0.6%)	602 (12.1%)	529 (10.6%)	16 (0.5%)	188 (9.8%)	46 (7.3%)	1,414 (6.80)
*Candida* spp.	43 (0.8%)	245 (4.9%)	582 (11.7%)	57 (1.8%)	48 (2.5%)	19 (3.0%)	994 (4.70)
*Enterobacter* spp.	287 (5.4%)	192 (3.9%)	104 (2.1%)	147 (4.7%)	86 (4.5%)	13 (2.1%)	829 (4.00)
*Streptococcus* spp.	45 (0.8%)	61 (1.2%)	34 (0.7%)	49 (1.6%)	19 (1.0%)	19 (3.0%)	227 (1.10)
*Stenotrophomonas maltophilia*	28 (0.5%)	143 (2.9%)	3 (0.1%)	32 (1.0%)	19 (1.0%)	1 (0.2%)	226 (1.10)
*Proteus* spp.	41 (0.8%)	24 (0.5%)	77 (1.5%)	31 (1.0%)	16 (0.8%)	3 (0.5%)	192 (0.90)
*Citrobacter* spp.	46 (0.9%)	27 (0.5%)	49 (1.0%)	3 (0.1%)	3 (0.2%)	5 (0.8%)	133 (0.60)
*Sarashia* spp.	22 (0.4%)	39 (0.8%)	9 (0.2%)	22 (0.7%)	21 (1.1%)	7 (1.1%)	120 (0.60)
*Burkholderia* spp.	1 (0.0%)	2 (0.0%)	13 (0.3%)	1 (0.0%)	1 (0.1%)	1 (0.2%)	19 (0.10)
*Salmonella* spp.	0	6 (0.1%)	0	0	0	2 (0.3%)	8 (0)
*Micrococcus* spp.	1 (0.0%)	5 (0.1%)	0	0	1 (0.1%)	1 (0.2%)	8 (0)
*Bacillus anthracis*	2 (0.0%)	0	1 (0.0%)	0	0	0	3 (0)
*Shigella* spp.	0	1 (0.0%)	0	0	1 (0.1%)	1 (0.2%)	3 (0)
*Clostridium perfringens*	2 (0.0%)	0	1 (0.0%)	0	0	0	3 (0)
*Clostridium difficile*	0	0	0	0	0	2 (0.3%)	2 (0)
*Corynebacterium diphtheria*	0	2 (0.0%)	0	0	0	0	2 (0)
*Morganella* spp.	0	0	0	0	1 (0.1%)	1 (0.2%)	2 (0)
*Aspergillus* spp.	0	0	0	1 (0.1%)	1 (0.1%)	0	2 (0)
*Mucormycosis* spp.	0	0	0	0	1 (0.1%)	1 (0.2%)	2 (0)
*Bacillus cereus*	0	0	0	0	1 (0.1%)	0	1 (0)

Numbers are presented as *N* (%).

VAE, Ventilator-associated event; BSI, Bloodstream infection; UTI, Urinary tract infection; PMEU, Pneumonia; SSI, Surgical Site infection.

When comparing the frequency of isolated microorganisms between pre-COVID-19 and COVID-19 era as depicted in Table 3, there was a significant difference in frequency of the following 10 microorganisms, while no statistical difference was found in the rest; *Klebsiella* species (23.2% vs. 29.4%), *Acinetobacter* species (17.9% vs. 21.7%), Enterococcus species (6.1% vs. 7.3%), *Escherichia coli* (13.6% vs. 11.8%), *Staphylococcus* species (14.7% vs. 9.8%), *Candida* species (6.4% vs. 3.2%), *Streptococcus* species (1.4% vs. 0.8%), *Stenotrophomonas maltophilia* (1.3% vs. 0.9%), *Proteus* species (1.1% vs. 0.7%), and *Serratia* species (0.8% vs. 0.3%). There was a statistically significant increase in the first three microorganisms and a decrease in the seven following.

**Table 3:** Comparison of the frequency of isolated microorganisms between COVID-19 and pre-COVID-19 era.

	Before COVID-19 era (03/21/2017 to 12/31/2019)	Within COVID-19 era (01/01/2020 to 09/22/2022)	Total
*Klebsiella* spp.^*^	2,296 (23.2%)	3,246 (29.4%)	5,542 (26.50)
*Acinetobacter* spp.^*^	1,776 (17.9%)	2,395 (21.7%)	4,171 (19.90)
Escherichia coli^*^	1,353 (13.6%)	1,298 (11.8%)	2,651 (12.70)
*Staphylococcus* spp.^*^	1,462 (14.7%)	1,075 (9.8%)	2,537 (12.10)
*Pseudomonas aeruginosa*	844 (8.5%)	1,007 (9.1%)	1,851 (8.80)
*Enterococcus* spp.^*^	604 (6.1%)	810 (7.3%)	1,414 (6.80)
*Candida* spp.^*^	637 (6.4%)	357 (3.2%)	994 (4.70)
*Enterobacter* spp.	389 (3.9%)	440 (4.0%)	829 (4.00)
*Streptococcus* spp.^*^	139 (1.4%)	88 (0.8%)	227 (1.10)
Stenotrophomonas maltophilia^*^	127 (1.3%)	99 (0.9%)	226 (1.10)
*Proteus* spp.^*^	113 (1.1%)	79 (0.7%)	192 (0.90)
*Citrobacter* spp.	73 (0.7%)	60 (0.5%)	133 (0.60)
*Sarashia* spp.^*^	83 (0.8%)	37 (0.3%)	120 (0.60)
*Burkholderia* spp.	5 (0.1%)	14 (0.1%)	19 (0.10)
*Salmonella* spp.	4 (0.0%)	4 (0.0%)	8 (0)
*Micrococcus* spp.	3 (0.0%)	5 (0.0%)	8 (0)
*Bacillus anthracis*	1 (0.0%)	2 (0.0%)	3 (0)
*Shigella* spp.	3 (0.0%)	0	3 (0)
*Clostridium perfringens*	0	3 (0.0%)	3 (0)
*Clostridium difficile*	1 (0.0%)	1 (0.0%)	2 (0)
*Corynebacterium diphtheria*	2 (0.0%)	0	2 (0)
*Morganella* spp.	1 (0.0%)	1 (0.0%)	2 (0)
*Aspergillus* spp.	0	2 (0.0%)	2 (0)
*Mucormycosis* spp.	0	2 (0.0%)	2 (0)
*Bacillus cereus*	1 (0.0%)	0	1 (0)
Total	9,917 (100%)	11,025 (100%)	20,942

* There was a statistically significant difference in the frequency of culture species between COVID-19 and the pre-COVID-19 era at a level of 0.05.

**Figure 2:**
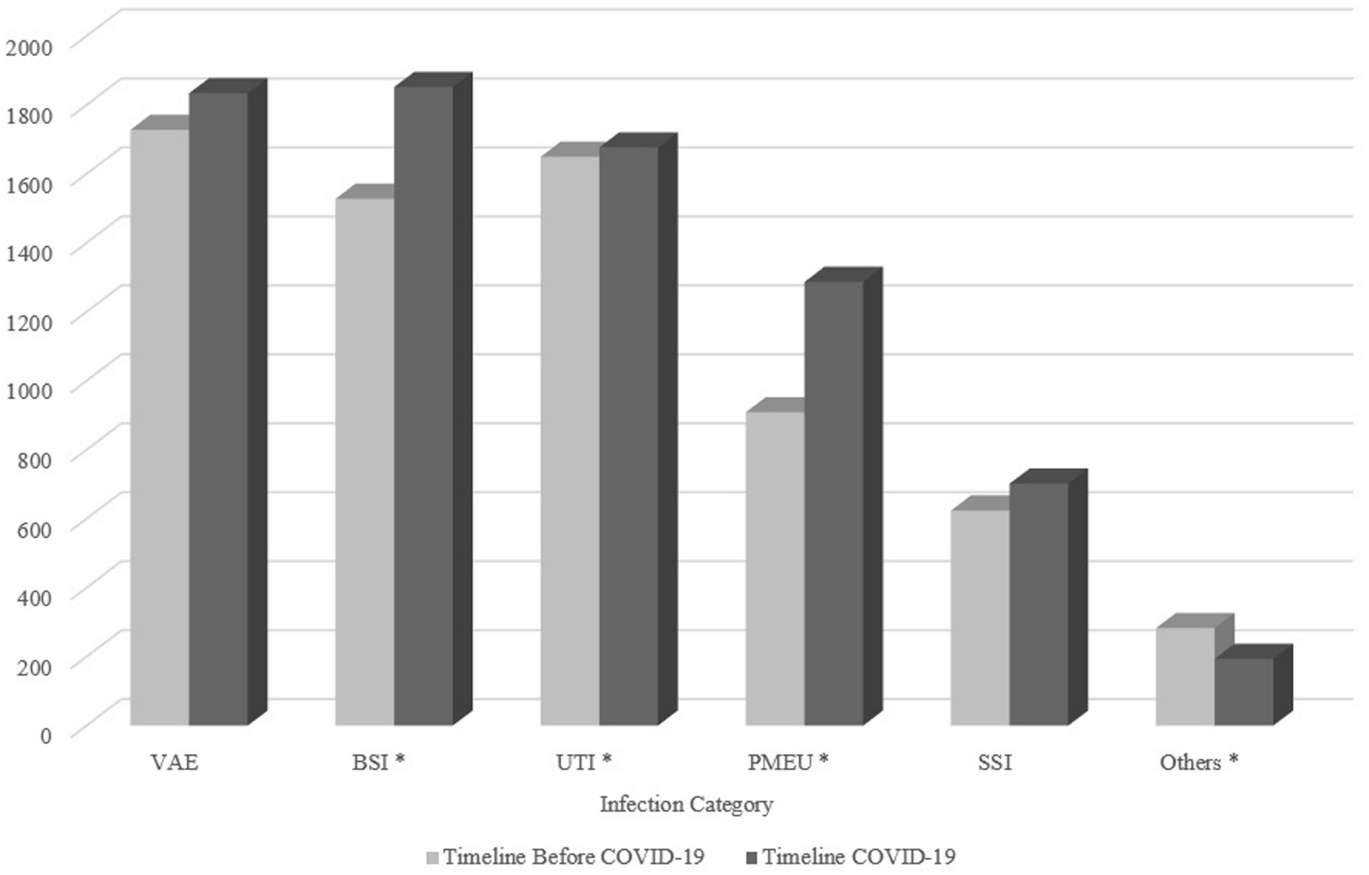
The pattern of infection categories before and during COVID-19. The asterisk demonstrates infection categories with a significant difference between two timelines. VAE, Ventilator-associated event; BSI, Bloodstream infection; UTI, Urinary tract infection; PMEU, Pneumonia; SSI, Surgical site infection.

As shown in Table 4, of the six main infection categories, there was a statistically significant difference in UTI, PMEU, BSI, and others, with an increase in PMEUs (13.5% vs. 17.1%) and BSI (22.7% vs. 24.5%) and decrease in UTIs (24.5% vs. 22.2%) and others (4.2% vs. 2.6%) in COVID-19 era compared to pre-COVID-19. Figure 2 demonstrates the pattern of infection categories before and during COVID-19.

**Table 4:** Comparison of the frequency of six main infection categories between COVID-19 and pre-COVID-19 era.

	Before COVID-19 (03/21/2017 to 12/31/2019)	COVID-19 (01/01/2020 to 09/22/2022)	Total
VAE	1,728 (25.7)	1,834 (24.3)	3,562 (25)
BSI^*^	1,528 (22.7)	1,852 (24.5)	3,380 (23.7)
UTI^*^	1,650 (24.5)	1,677 (22.2%)	3,327 (23.3)
PMEU^*^	909 (13.5)	1,287 (17.1)	2,196 (15.4)
SSI	624 (9.3)	702 (9.3)	1,326 (9.3)
Others^*^	283 (4.2)	194 (2.6)	477 (3.3)
Total	6,722 (100)	7,546 (100)	14,268 (100)

* There was a statistically significant difference in the frequency of infection categories between COVID-19 and pre-COVID-19 era at a level of 0.05 (*p*-values < 0.001).

VAE, Ventilator-associated event; BSI, Bloodstream infection; UTI, Urinary tract infection; PMEU, Pneumonia; SSI, Surgical Site infection.

As depicted in Table 5, the odds of in-hospital mortality in all six main infection categories have been significantly increased in the COVID-19 era, when compared to the pre-COVID-19 era. The odds of death were increased in the SSI group more than the rest (OR:2.65, CI 95%: 2.13–3.29). even after adjustment for age, gender, and underlying disorder of cases, the odds of in-hospital mortality still increased significantly in the COVID-19 era, compared to pre-COVID-19. Likewise, the odds of death were the highest in the SSI group (aOR: 2.55, CI 95%: 1.62–4.00).

**Table 5:** Final crude and multiple logistic regression model of each infectious category and in-hospital mortality, while comparing the COVID-19 era with the pre-COVID-19 era (as the reference group).

	Model 1			Model 2^*^			Total
	OR	CI 95%	*p*-value	aOR	CI 95%	*p*-value	
VAE	1.50	1.35–1.68	<0.001	1.57	1.40–1.75	<0.001	5,542 (26.50)
BSI	1.61	1.43–1.81	<0.001	1.87	1.65–2.12	<0.001	4,171 (19.90)
UTI	1.59	1.41–1.80	<0.001	1.80	1.58–2.04	<0.001	2,651 (12.70)
PMEU	1.90	1.63–2.23	<0.001	1.92	1.64–2.26	<0.001	2,537 (12.10)
SSI	2.65	2.13–3.29	<0.001	2.98	2.37–3.74	<0.001	1,851 (8.80)
Others	1.97	1.29–3.00	0.002	2.55	1.62–4.00	<0.001	1,414 (6.80)

* Adjusted for age, sex, underlying disorders.

VAE, Ventilator-associated event; BSI, Bloodstream infection; UTI, Urinary tract infection; PMEU, Pneumonia; SSI, Surgical Site infection.

As demonstrated in Table 6, there was a heterogenous trend for different six main infection categories regarding the prolonged ICU LOS outcomes. Alteration in the COVID-19 era, compared to pre-COVID-19 was statistically significant in only two groups; BSI and PMEU. The odds of prolonged ICU LOS were decreased by 33% in the BSI group in the COVID-19 era (OR: 0.67, CI 95%: 0.55–0.82), while increased by 47% in the PMEU group in the COVID-19 era (OR: 1.47, CI 95%: 1.14–1.90). the results remained still significant after controlling for age, gender, and underlying disorders (aORs: 0.67 for BSI and 1.41 for PMEU).

**Table 6:** Final crude and multiple logistic regression model of each infectious category and prolonged ICU stay, while comparing the COVID-19 era with the pre-COVID-19 era (as the reference group).

	Model 1			Model 2^*^			Total
	OR	CI 95%	*p*-value	aOR	CI 95%	*p*-value	
VAE	0.91	0.72–1.14	0.407	0.91	0.73–1.15	0.444	5,542 (26.50)
BSI	0.67	0.55–0.82	<0.001	0.67	0.55–0.83	<0.001	4,171 (19.90)
UTI	1.01	0.86–1.19	0.886	0.97	0.82–1.14	0.691	2,651 (12.70)
PMEU	1.47	1.14–1.90	0.003	1.41	1.09–1.83	0.008	2,537 (12.10)
SSI	1.01	0.69–1.47	0.949	0.99	0.67–1.46	0.95	1,851 (8.80)
Others	0.84	0.48–1.47	0.553	0.78	0.44–1.37	0.510	1,414 (6.80)

* Adjusted for age, sex, underlying disorders.

VAE, Ventilator-associated event; BSI, Bloodstream infection; UTI, Urinary tract infection; PMEU, Pneumonia; SSI, Surgical site infection.

## 4 Discussion

This study investigated the frequency of HAIs in ICUs of all government hospitals affiliated with the Tehran University of Medical Sciences from 21st March 2017 to 22nd September 2022. The first officially reported cases of COVID-19 in Iran were on February 19, 2020 (15). In our study, we defined the pandemic period as the time from March 2020, to September 2022 and March 2017 to the end February 2020 as pre-pandemic period. The main objective was to examine the frequency of different types of hospital-acquired isolated microorganisms in ICUs and to investigate the most common type of microorganism each year. Furthermore, the study provides data on the frequency of different HAI categories, which can inform the development of appropriate treatment and prevention strategies. In this study, *Klebsiella* species were the most frequently isolated microorganism during the COVID-19 era, consistent with our previous findings (16). For instance, we realized VAE was the most common type of HAI category. The data can be used to monitor the effectiveness of infection control measures and guide future research in this area. Afterward, we compared the outcomes of patients with each infection category in the COVID-19 and pre-COVID-19 era.

There have been several studies conducted on the frequency of HAIs in ICUs in different regions and countries. A comparison of these studies with the results of the current study can provide a broader perspective on the issue. In a single-center study of HAIs in ICU-admitted patients in Spain during the COVID-19 pandemic, 57 cases developed a microbial infection. BSIs were the most common infection category reported in 56%, followed by VAEs in 23%, hospital-acquired pneumonia in 10%, UTI in 8%, and soft tissue in 2% (17).

In a single center study in Iran, comparing HAI trends within COVID-19 and the pre-COVID-19 era revealed that VAEs were reduced within the COVID-19 era. Despite being statistically insignificant in our study, the results were similar. In contrast to our findings, they reported a significant decrease in *Acinetobacter* species frequency in the COVID-19 era, when compared to pre-COVID-19 (18).

In 2020, there was a notable surge in the national rates of central-line associated BSI (CLABSI), Catheter-associated UTI (CAUTI), VAE, and Methicillin-resistant *Staphylococcus aureus* (MRSA) bacteremia, with the most significant increase observed in CLABSI cases. This rise in device-associated infections during the pandemic was attributed to various factors, including the prolonged hospital stays of patients, the admission of more critically ill individuals with multiple comorbidities, and the extended use of medical devices. Additionally, researchers noted a heightened occurrence of VAEs among critically ill COVID-19 patients. In light of these findings, they emphasized that maintaining traditional infection prevention and control measures is imperative to mitigate the spread of infections in healthcare settings (19, 20). Their results contradict our findings. Here, we showed that there was an increase in ICU LOS during the pandemic, which might be due to significantly decreased patients’ ICU survival during the pandemic that might decrease LOS. Besides, due to overcrowding of intensive units during the pandemic, the interest was toward to early transfer of patients to other hospital wards. BSIs were similarly increased in our study. An insignificant decrease in VAEs during the pandemic in this study might be to some degree due to the separation of PMEUs from VAEs.

In a single center study of 578 ICU-admitted cases in Malaysia, although statistically insignificant, an increase in the frequency of CLABSI and VAE as well as a decrease in pneumonia, UTI, and SSI were reported (21). Their findings for BSI and UTI were in parallel to ours. Overall, the comparison of these studies with the current study suggests that the nature of HAIs in ICUs varies across regions and countries, and the most common types of infections also differ. However, all studies emphasize the need for continuous monitoring and improvement of infection control measures in ICUs to reduce the incidence of HAIs and improve patient outcomes (22, 23).

There is a bulk of studies in the literature that evaluated the effect of HAI on in-hospital mortality during the COVID-19 pandemic. For instance, in a multicenter study in the UK, Khan et al. realized that nosocomial infections did not significantly increase the 30-day mortality in COVID-19 patients (24). On the contrary, before the emergence of vaccines in COVID-19, Dave et al. concluded that 30 days’ mortality significantly increased the 30 days’ mortality in COVID-19 cases.

Despite of considerable number of studies assessing the impact of HAIs on COVID-19 mortality, to the extent of our knowledge, there were no studies evaluating the impact of COVID-19 on HAI mortality, when compared to the pre-COVID-19 era. This study also reported an increase in the mortality rate attributed to HAIs during the COVID-19 pandemic compared to the pre-COVID period. This finding underscores the need for increased attention to infection control measures in ICUs, especially during pandemics, to prevent HAIs and reduce mortality rates.

## 5 Study limitations

We postulated that COVID-19-induced pneumonia could have been a significant reason for ICU admissions, thereby potentially altering microorganisms’ distribution. However, due to the system-based nature of the patient data in this study, we unfortunately do not have access to the specific indications for ICU admission.

## 6 Conclusion

COVID-19 changed the pattern of HAIs and also increased their mortality. Overall, the findings of this study emphasize the importance of continuous monitoring and improvement of infection control measures in ICUs to reduce the incidence of HAIs and improve patient outcomes.

## Data Availability

The raw data supporting the conclusions of this article will be made available by the authors without undue reservation.

## References

[1] Centers for Disease Control and Prevention. Identifying healthcare-associated infections (HAI) for NHSN surveillance. In: National Healthcare Safety Network (NHSN) Patient Safety Component Manual, Chapter 2. Atlanta (GA): National Center for Emerging and Zoonotic Infectious Diseases, Division of Healthcare Quality Promotion. (2025) Available at: https://www.cdc.gov/nhsn/PDFs/pscManual/2PSC_IdentifyingHAIs_NHSNcurrent.pdf

[2] Savey A Lepape A Palomar M Agodi A Hiesmayr M Magiorakos AP. Surveillance of healthcare-associated infections and prevention indicators in European intensive care units: HAI-Net ICU protocol, version 2.2. Stockholm: European Centre for Disease Prevention and Contro. (2017). Available at: https://www.ecdc.europa.eu/sites/default/files/documents/HAI-Net-ICU-protocol-v2.2_0.pdf

[3] Vokes RA Bearman G Bazzoli GJ. Hospital-acquired infections under pay-for-performance systems: an administrative perspective on management and change. Curr Infect Dis Rep. (2018) 20:1–7. doi: 10.1007/s11908-018-0638-5, PMID: 30051191

[4] Vincent J-L Bihari DJ Suter PM Bruining HA White J Nicolas-Chanoin M-H. The prevalence of nosocomial infection in intensive care units in Europe: results of the European prevalence of infection in intensive care (EPIC) study. JAMA. (1995) 274:639–44. doi: 10.1001/jama.1995.03530080055041 7637145

[5] European Centre for Disease Prevention and Control. Healthcare-associated infections acquired in intensive care units annual epidemiological report for 2019. Stockholm: ECDC; (2023). Available at: https://www.ecdc.europa.eu/sites/default/files/documents/healthcare-associated-infections-intensive-care-units-annual-epidemiological-report-2019.pdf?utm_source=chatgpt.com

[6] Önal U Tüzemen Ü Kazak E Gençol N Souleiman E İmer H. Effects of COVID-19 pandemic on healthcare-associated infections, antibiotic resistance and consumption rates in intensive care units. Infez Med. (2023) 31:195–203. doi: 10.53854/liim-3102-7, PMID: 37283640 PMC10241394

[7] Bennett EE VanBuren J Holubkov R Bratton SL. Presence of invasive devices and risks of healthcare-associated infections and sepsis. J Pediatr Intensive Care. (2018) 7:188–95. doi: 10.1055/s-0038-1656535, PMID: 31073493 PMC6506685

[8] Haque M McKimm J Sartelli M Dhingra S Labricciosa FM Islam S. Strategies to prevent healthcare-associated infections: a narrative overview. Risk Manage Healthcare Policy. (2020) 13:1765–80. doi: 10.2147/RMHP.S269315, PMID: 33061710 PMC7532064

[9] Tosi M Roat E de Biasi S Munari E Venturelli S Coloretti I. Multidrug resistant bacteria in critically ill patients: a step further antibiotic therapy. J Emerg Crit Care Med. (2018) 2:2. doi: 10.21037/jeccm.2018.11.08, PMID: 39866280

[10] Vaillancourt M Jorth P. The unrecognized threat of secondary bacterial infections with COVID-19. MBio. (2020) 11:e01806–20. doi: 10.1128/mBio.01806-20, PMID: 32769090 PMC7419722

[11] Chen X Liao B Cheng L Peng X Xu X Li Y. The microbial coinfection in COVID-19. Appl Microbiol Biotechnol. (2020) 104:7777–85. doi: 10.1007/s00253-020-10814-6, PMID: 32780290 PMC7417782

[12] Zhou F Yu T du R Fan G Liu Y Liu Z. Clinical course and risk factors for mortality of adult inpatients with COVID-19 in Wuhan, China: a retrospective cohort study. Lancet. (2020) 395:1054–62. doi: 10.1016/S0140-6736(20)30566-3, PMID: 32171076 PMC7270627

[13] Alhumaid S al Mutair A al Alawi Z Alshawi AM Alomran SA Almuhanna MS. Coinfections with bacteria, fungi, and respiratory viruses in patients with SARS-CoV-2: a systematic review and meta-analysis. Pathogens. (2021) 10:809. doi: 10.3390/pathogens10070809, PMID: 34202114 PMC8308492

[14] Centers for Disease Control and Prevention (CDC). Interim guidelines for collecting and handling of clinical specimens for COVID-19 testing. (2019). Available at: https://www.cdc.gov/coronavirus/2019-ncov/lab/guidelines-clinical-specimens.html

[15] Yavarian J Shafiei-Jandaghi NZ Sadeghi K Shatizadeh Malekshahi S Salimi V Nejati A. First cases of SARS-CoV-2 in Iran, 2020: case series report. Iran J Public Health. (2020) 49:1564–8. doi: 10.18502/ijph.v49i8.3903, PMID: 33083334 PMC7554384

[16] Khavandegar A Siami Z Goudarzi S Rasooli A Ettehad Y. Investigation of microbial coinfection in 453 septic COVID-19 patients admitted to hospital; a retrospective study. Future Sci OA. (2023) 9:p. FSO884. doi: 10.2144/fsoa-2023-0066, PMID: 37752919 PMC10518821

[17] Bardi T Pintado V Gomez-Rojo M Escudero-Sanchez R Azzam Lopez A Diez-Remesal Y. Nosocomial infections associated to COVID-19 in the intensive care unit: clinical characteristics and outcome. Eur J Clin Microbiol Infect Dis. (2021) 40:495–502. doi: 10.1007/s10096-020-04142-w, PMID: 33389263 PMC7778834

[18] Zand F Vakili H Asmarian N Masjedi M Sabetian G Nikandish R. Unintended impact of COVID-19 pandemic on the rate of catheter related nosocomial infections and incidence of multiple drug resistance pathogens in three intensive care units not allocated to COVID-19 patients in a large teaching hospital. BMC Infect Dis. (2023) 23:11. doi: 10.1186/s12879-022-07962-7, PMID: 36609225 PMC9821351

[19] al-Hasan M Winders H Bookstaver P Justo J. Direct measurement of performance: a new era in antimicrobial stewardship. Antibiotics. (2019) 8:127. doi: 10.3390/antibiotics8030127, PMID: 31450576 PMC6784134

[20] Leblebicioglu H Erben N Rosenthal VD Atasay B Erbay A Unal S. International nosocomial infection control consortium (INICC) national report on device-associated infection rates in 19 cities of Turkey, data summary for 2003–2012. Ann Clin Microbiol Antimicrob. (2014) 13:1–13. doi: 10.1186/s12941-014-0051-3 25403704 PMC4255447

[21] Ismaeil R Nahas ARF Kamarudin NB Abubakar U Mat-Nor MB Mohamed MHN. Evaluation of the impact of COVID-19 pandemic on hospital-acquired infections in a tertiary hospital in Malaysia. BMC Infect Dis. (2023) 23:779. doi: 10.1186/s12879-023-08770-3, PMID: 37946158 PMC10636815

[22] Tabah A Koulenti D Laupland K Misset B Valles J Bruzzi de Carvalho F. Characteristics and determinants of outcome of hospital-acquired bloodstream infections in intensive care units: the EUROBACT international cohort study. Intensive Care Med. (2012) 38:1930–45. doi: 10.1007/s00134-012-2695-9, PMID: 23011531

[23] McFee RB. Nosocomial or hospital-acquired infections: an overview. Dis Mon. (2009) 55:422–38. doi: 10.1016/j.disamonth.2009.03.014, PMID: 19540995 PMC7094512

[24] Khan KS Reed-Embleton H Lewis J Saldanha J Mahmud S. Does nosocomial COVID-19 result in increased 30-day mortality? A multi-Centre observational study to identify risk factors for worse outcomes in patients with COVID-19. J Hosp Infect. (2021) 107:91–4. doi: 10.1016/j.jhin.2020.09.017, PMID: 32950587 PMC7495174

